# Exploring the impact of chronic intermittent EU-GMP certified *Cannabis sativa* L. therapy and its relevance in a rat model of aging

**DOI:** 10.1186/s42238-025-00313-8

**Published:** 2025-08-06

**Authors:** Ivona-Maria Tudorancea, Gabriela-Dumitrita Stanciu, Carmen Solcan, Mitica Ciorpac, Andrei Szilagyi, Daniela-Carmen Ababei, Raluca-Maria Gogu, Bogdan-Ionel Tamba

**Affiliations:** 1 Advanced Research and Development Center for Experimental Medicine ”Prof, Ostin C. Mungiu” - CEMEX, “Grigore T. Popa” University of Medicine and Pharmacy of Iasi, Iasi, Romania; 2 Faculty of Veterinary Medicine, Ion Ionescu de La Brad, University of Life Sciences, Iasi, Romania; 3 Pharmacodynamics and Clinical Pharmacy Department, “Grigore T. Popa” University of Medicine and Pharmacy of Iasi, Iasi, Romania; 4 Department of Pharmacology, Clinical Pharmacology and Algesiology, “Grigore T. Popa” University of Medicine and Pharmacy of Iasi, Iasi, Romania

**Keywords:** *Cannabis sativa* L., EU-GMP certification, Chronic intermittent therapy, Aging, Rat model, Neuroprotection

## Abstract

**Background:**

Aging is a multifaceted process marked by the progressive accumulation of cellular damage in various tissues, resulting in a decline in physiological functions. The primary aim of aging research is to identify compounds that can delay or mitigate these detrimental changes. As cannabis legalization becomes more widespread and with limited empirical studies on its effects in the aging human population, there is a pressing need for research into the impact of Cannabis and cannabinoids on healthy aging and age-related diseases.

**Methods:**

Our study aims to evaluate the effects of chronic, intermittent exposure, defined as 6 weeks of use of EU-GMP certified *Cannabis sativa* L. (Cannabixir® Medium Flos) administration, dosed at 6.25 and 25 mg/kg on neurobiological changes in naturally aged rats and its potential efficacy in mitigating age-related alterations. The impact of the Cannabixir® Medium Flos was assessed through clinical, histopathological, immunohistochemical, and behavioral evaluations.

**Results:**

Cannabixir® Medium Flos was found to be generally safe, with no significant effects on motor performance and a neutral effect on anxiety-like behavior. Histological analysis revealed that the hippocampus of aged rats treated with this compound—an area known for its abundance of endocannabinoids and cannabinoid receptor type 1—exhibited characteristics similar to those observed in young adult rats. Additionally, the study suggests that chronic, intermittent treatment with Cannabixir® Medium Flos may modulate astrocyte function, reduce neuroinflammation, and potentially influence cell proliferation and neuronal apoptosis in a dose-dependent manner. However, these preliminary findings should be interpreted with caution, as the study's exploratory nature.

**Conclusions:**

These preliminary findings suggest that cannabinoid therapy targeting the endocannabinoid system may offer potential neuroprotective benefits in aging. While the study offers valuable preclinical insights into the effects of an EU-GMP-certified cannabinoid receptor ligand in reducing age-related cognitive decline, these effects are likely mediated by a combination of mechanisms. Given the complex phytochemical composition, the observed outcomes cannot be attributed exclusively to cannabinoid receptor activation. Accordingly, these findings should be interpreted with caution, and further studies employing more targeted methodologies are needed to elucidate the underlying mechanisms.

## Introduction

In the past century, the global demographic profile has undergone profound changes, most notably a significant rise in the proportion of older adults. By the late 2070 s, the number of people aged 65 and over is expected to reach 2.2 billion globally, outnumbering those under the age of 18 (United Nations, 2024).

Aging is commonly defined as the progressive loss of physiological integrity over time, leading to functional decline and greater vulnerability to disease and death (Tabula Muris, 2020). This multifactorial process affects multiple organ systems and contributes to age-related phenotypes, including cognitive decline (World Health Organization, 2023) Neuroinflammation, oxidative stress, immunosenescence and physical frailty further exacerbate these changes, underscoring the integrative nature of aging (Giaimo and Traulsen 2022). 

Recent preclinical and clinical studies indicate age-related alterations in the endocannabinoid system (ECS), highlighting its key role in aging and associated disorders (Paradisi, et al., 2006). The ECS modulates neuronal and glial function, exerts neuroprotective effects and regulates neuroinflammation and stress responses (Fogaca et al., 2018; Maldonado et al. 2020; Ruiz de Martín et al., 2022). The ECS comprises two primary receptor subtypes—CB1 and CB2—alongside endogenous ligands and the enzymes involved in their synthesis and degradation (Abate et al. 2021). Current research employs cannabinoid receptor ligands, such as ∆9-tetrahydrocannabinol (THC) and cannabidiol (CBD), to elucidate the role of the ECS in age-related pathologies (Faria et al. 2020; de Almeida et al. 2021; Nitzan et al. 2022). Under inflammatory conditions, CB2 receptors regulate glial activity (Komorowska-Müller et al. 2021; Ruiz de Martín et al., 2022) and promote pro-inflammatory cytokine production (Song and Colonna 2018). Inflammaging, a hallmark of aging, is marked by chronic low-grade inflammation and elevated levels of cytokines. Although its mechanisms are not fully understood, modulating age-related inflammatory pathways may offer therapeutic benefits for older adults (Franceschi and Campisi 2014; Rea et al. 2018).

Neuroinflammatory processes contribute to both normal aging and neurodegenerative diseases, accompanied by changes in ECS expression marked by reduced CB1 receptor levels in neurons and increased CB2 expression in activated microglia (Komorowska-Müller et al. 2021; Ruiz de Martín et al., 2022). In Alzheimer’s disease (AD), CB1 expression is elevated in early stages but declines as pathology advances, suggesting a potential modulatory role (Ramírez et al., 2005). In contrast, CB2 upregulation in AD-associated microglia correlates with amyloid-β burden and plaque density (Benito et al. 2003). Moreover, CB1 knockout models show early cognitive impairment and accelerated neurodegenerative markers, supporting ECS involvement in brain aging (Palmisano et al., 2023). Despite encouraging preclinical findings, clinical evidence for cannabinoid efficacy in neurodegenerative conditions remains limited (Stanciu et al. 2024; Erridge et al. 2024).

Understanding the ECS’s role in aging could lead to therapies that boost resilience, prevent age-related diseases and enhance well-being. To this end, our research focused on assessing the effects of chronic, intermittent exposure to Cannabixir® Medium Flos—a European Union Good Manufacturing Practice (EU-GMP) certified *Cannabis sativa* L. inflorescence with 15.6% THC and < 1% CBD, on neurobiological changes in naturally aged, healthy rats. The selection of Cannabixir® as the study drug was guided by scientific evaluation and the need to address knowledge gaps in cannabinoid research. Chosen for its favorable pharmacokinetic profile, Cannabixir® has been thoroughly evaluated in prior toxicity studies by our team (Filipiuc et al. 2023). The product also meets EU-GMP quality standards, ensuring consistency and suitability for reproducible medical applications. Conversely, this study provides new insights into the pharmacological profiles of THC precursors, particularly ∆9-tetrahydrocannabinolic acid (THCA-A) and cannabigerolic acid (CBGA). THCA-A is particularly notable in preclinical studies for its non-psychotropic profile and demonstrated neuroprotective and anti-inflammatory effects (Palomares et al. 2020). Additional evidence supports its role in reducing adipose tissue, modulating obesity-related metabolic dysfunction and alleviating nausea (Nadal et al. 2017; Palomares et al. 2020; Rock et al. 2013). However, its effects in aging models remain unexplored. THCA-A's physicochemical instability, particularly its propensity to decarboxylate into THC, is a known limitation. In this aging animal model, we assessed THCA-A within a phytocannabinoid complex, given that terpenes in the Cannabis phytocomplex possess antioxidant activity known to inhibit its oxidative decarboxylation into THC (Morano et al. 2022). Our research simultaneously aims to evaluate the behavioral effects of an original Cannabis strain and to explore strategies to mitigate the “on-target” side effects typically observed in chronic Cannabis use. Due to its exploratory nature, the study focused on qualitative histological observations to identify potential tissue-level trends. Preliminary IHC analysis was performed to assess changes in hippocampal neurogenesis and neuroinflammation, based on representative samples selected by the anatomist-pathologist. These findings were complemented by peripheral cytokine screening to detect markers of chronic low-grade inflammation. Overall, this work serves as a preliminary investigation toward advancing cannabinoid-based therapeutics and addressing aging-related pathobiological changes.

## Methods

### Animal care

In this study, male Sprague–Dawley rats, aged 8 months (young adult) and 19 months (naturally aged), from the Cantacuzino Institute, Romania, were used. The rats were housed in individually ventilated cages (IVCs) at the animal facility of the Advanced Research and Development Center for Experimental Medicine,"Prof. Ostin C. Mungiu"—CEMEX. They were maintained under standard husbandry conditions, including controlled room temperature (20 ± 4 °C), relative humidity (50 ± 5%), and a controlled 12:12 h light–dark cycle, with unrestricted access to water and standard laboratory chow. The experimental protocols and procedures were carried out at CEMEX in accordance with European Community Guidelines (Directive 2010/63/EU) and Romanian legislation (Law no. 43/2014) on the protection of animals used for scientific purposes. These protocols were rigorously reviewed and approved by both the Ethical Committee of the “Grigore T. Popa” University of Medicine and Pharmacy of Iasi (approval no. 343/07.09.2023) and the Romanian National Sanitary Veterinary and Food Safety Authority (approval no. 68/06.11.2023).

### Reagents

The *Cannabis sativa* L. phytocomplex (Cannabixir® Medium Flos—PZN: 7,001,905; Cansativa GmbH, Mörfelden-Walldorf, Germany) used in this study was certified in accordance with EU-GMP standards and contained 15.6% THC and < 1% CBD, as reported in the manufacturer’s Certificate of Analysis No. POO5840/11.05.2022. The batch of medicinal products was manufactured in accordance with EU Good Manufacturing Practice, the EU Pharmacopeia and the Notice on the German Pharmacopeia 2017 issued by the German Federal Institute for Drugs and Medical Devices on May 5, 2017. The testing was carried out in a facility with a valid permit in accordance with Sect. 13 of the German Medicinal Products Act (AMG) – Table 1.

**Table 1:** Quality control parameters of the *Cannabis sativa* L. phytocomplex

Test	Analytical methods	Specifications	Measured value/Complies
Properties			
Smell	DAB Monograph Cannabis Inflorescences	Characteristic of Cannabis inflorescences	Characteristic of Cannabis inflorescences
Identity verification			
Identification A (macroscopic) Identification B (microscopic)	Identification test for Cannabis A inflorescences from the DAB monograph	Complies with the description	Complies with the description
Identification C (DC)	Ph. Eur. 2.2.27 and DAB Monograph for Cannabis Inflorescences	Complies with the description	Complies with the description
Purity			
Foreign matter	Ph. Eur. 2.8.2 and DAB Monograph for Cannabis Inflorescences	Max 2% (m/m)	< 2% (m/m)
Loss on drying	Ph. Eur. 2.2.32	Max 10% (m/m)	< 10% (m/m)
Cannabinol (HPLC)	Ph. Eur. 2.2.29	Max 1,0%	< 1,0%
Pesticide residues	Ph. Eur. 2.8.13	Shall comply	Complies
Heavy metals in herbal drugs and herbal drug preparations: • Cadmium (Cd) • Lead (Pb) • Mercury (Hg) • Arsenic (Ar)	Ph. Eur. 2.4.27	Shall comply • Max 1,0 ppm • Max 5,0 ppm • Max 0,1 ppm • Max 2 ppm	Complies: < 1,0 ppm < 5,0 ppm < 0,1 ppm < 2 ppm
Determination of aflatoxin B1 in herbal drugs Aflatoxin B1	Ph. Eur. 2.8.18	Max 2 µg/kg	< 2 µg/kg
Aflatoxins B1 + G1 + B2 + G2		Max 4 µg/kg	< 4 µg/kg
TAMC (Total aerobic microbial count)	Ph. Eur. 2.6.12	Max 104 CFU/g Max 50 000 CFU/g	< 104 CFU/g
TYMC (Total combined yeasts/moulds count)	Ph. Eur. 2.6.12	Max 102 CFU/g Max 500 CFU/g	< 102 CFU/g
bile salt-tolerant, gram-negative bacteria	Ph. Eur. 2.6.31	Max 102 CFU/g	< 102 CFU/g
Escherichia coli	Ph. Eur. 2.6.31	Not detected in 1 g	Negative in g
Salmonella sp.	Ph. Eur. 2.6.31	Not detected in 1 g	Negative in g

The dried inflorescence (Cannabixir® Medium Flos, PZN: 7,001,905; Cansativa GmbH, Mörfelden-Walldorf, Germany), was finely ground using an electrical mortar grinder RM 200 (Retsch GmbH, Haan, Germany), and then sieved through a 125-micron strainer (BSS Mesh No. 120). The resulting powder was dispersed in a 0.1% carboxymethyl cellulose sodium solution (CMC-Na). Cannabixir® Medium Flos (6.25 mg/kg and 25 mg/kg) and the 0.1% aqueous suspension of CMC-Na, as a vehicle, were administered via gavage at a volume adjusted to 0.5 mL/100 g of body weight. This dosing volume complies with standard safety recommendations for oral administration in rats, ensuring both appropriate weight-based dosing and minimal discomfort.

### Pharmacological treatment and study design

The doses selected for this study (6.25 mg/kg and 25 mg/kg) were carefully chosen to represent low and high doses within a therapeutic range, based on LD50 values, pharmacokinetic data, and safety information from our previous research (Filipiuc et al. 2023). This approach allowed us to evaluate dose-dependent effects while minimizing the risk of potential adverse effects. The dosing regimen was designed to ensure that THC levels remained below detectable thresholds within a 72-h period, thus minimizing any psychoactive effects and ensuring the safety of the treatment. Although a broader range of doses could provide additional insights, these two concentrations were selected to balance safety with the goal of assessing therapeutic effects in this preclinical model. Thirty-two rats were randomly assigned to four experimental groups (8 rats per group): the young adult control group (ADULT-Con), the naturally aged control group (OLD-Con), the Cannabixir® Medium Flos 6.25 mg/kg naturally aged group (OLD-6.25), and the Cannabixir® Medium Flos 25 mg/kg naturally aged group (OLD-25).

**Fig. 1:**
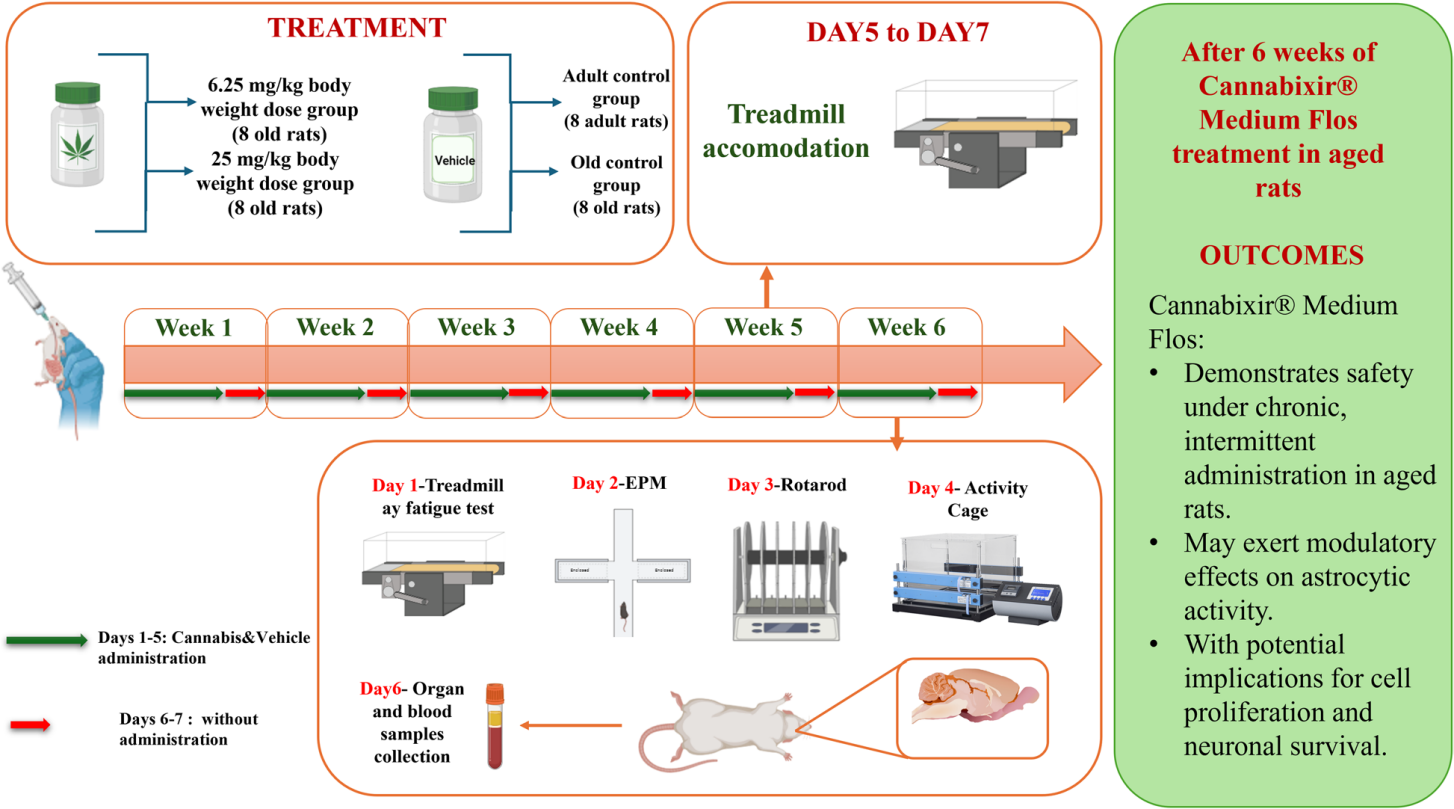
Study design

To model chronic intermittent cannabinoid exposure (Lamarque et al., 2001; Mouro et al. 2018), rats received daily administrations of Cannabis or vehicle, between 9:00 and 10:00 a.m. for 5 consecutive days, followed by two drug-free days, repeated over 6 weeks (Fig. 1). The dosing schedule was designed to maintain consistent exposure while minimizing variability in administration timing – an essential factor for accurately assessing behavioral and biological responses. Moreover, this approach aimed to mitigate potential"on-target"adverse effects associated with prolonged cannabinoid exposure from continuous administration. The 6-week treatment period was selected to model chronic cannabinoid exposure and the associated age-related changes within a biologically relevant timeframe for rats, which have a relatively short lifespan (2–3.5 years), compared to the global human life expectancy of approximately 80 years (Quinn 2005). Although lifespan-based conversion models (Sengupta 2013; Ghasemi et al. 2021) suggest that this 6-week period corresponds to 2.5–3.5 human years, we acknowledge that such extrapolations are approximate and depend on various factors, including pharmacokinetics, metabolism, and other species-specific variables. Thus, while these conversions offer a general guideline, the main objective of this study is not to directly equate the rodent treatment period with human aging but rather to gain insights into the long-term effects of cannabinoid exposure on aging processes within an animal model. These insights are crucial for developing strategies that may have therapeutic implications for human health, particularly in understanding the risks associated with chronic exposure.

Body weight was monitored weekly for each animal throughout the entire experimental period, starting from the day of treatment initiation (week 1) until the study endpoint (week 6). Animals were weighed on the same day of each week, specifically every Monday, to ensure consistency. To evaluate weight progression over time, weight variation was calculated as a percentage relative to the initial body weight. The following formula was used: Weight variation (%) = [(Wₙ − W₁)/W₁] × 100, where Wₙ represents the body weight in a given week and W₁ is the baseline weight recorded in week 1.

### Behavioral assessment of locomotion and anxiety-related responses

To evaluate locomotor and affective-like behavior, the Activity Cage, RotaRod, Treadmill, and Elevated Plus Maze (EPM) tests were employed. All behavioral assessments were conducted during the light phase under consistent environmental conditions, as previously described, to minimize potential circadian effects. Prior to testing, animals underwent a 3-day acclimation period to the experimental environment and, where applicable (specifically for the Treadmill), to the testing apparatus. This was done to reduce novelty-induced stress and ensure that observed behavioral changes reflected experimental manipulations rather than environmental unfamiliarity. The behavioral tests were performed according to a defined schedule: treadmill habituation occurred on days 5–7 of week 5, followed by testing in week 6, with treadmill on day 1, EPM on day 2, RotaRod on day 3, and Activity Cage on day 4. A minimum of 24 h of rest was allowed between tests. A detailed timeline of the schedule can be found in Fig. 1.

#### Assessing functional endurance in aging rodents using treadmill test

The Rat Preclinical Research Treadmill LE8700TS (Panlab/Harvard) was used following the experimental protocol: acclimatization of the rats in the running chamber for a period of 5 min, with an inclination of 0°, then each rat ran for 5 min with the speed of 10 m/min and an inclination of 0°. On the 2nd and 3rd days of training, the rats were allowed to run for 10 min at a 10° incline. An electrical stimulation (0.4 mA) on the metal grid behind the treadmill was applied. During the actual testing on the fourth day, the belt speed started at 10 m/min for a duration of 5 min, and it increased by 2 m/min every 2 min until it reached 46 m/min. Rats were allowed to run until exhaustion or until the treadmill reached maximum speed. The exhaustion was defined as 5 s of immobility with continuous stimulation. Work and power were calculated according to the formulas described by Lew et al., (2019) and showed below:

1. Work (J) = body mass (kg) × gravity (9.81 m/s2) × vertical speed (m/s × angle) × time (s)
2. Power (W) = Work (J)/time (s)

The value obtained from the Work parameter formula quantifies the mechanical work performed by the experimental animal on an inclined plane until its energy resources are fully depleted. The Power parameter reflects the mechanical power output, calculated as the ratio between the total work performed and the time taken to reach exhaustion.

#### Evaluation of anxiety-like behavior

For the EPM assessment, rats were placed in a maze shaped like a plus sign and elevated 50 cm above the ground. Their behavior was recorded for 5 min using the Smart 3.0 Basic Pack/Smart 3.0 SUPER video-tracking software (Harvard Apparatus, Holliston, MA, USA). For each animal, the raw number of entries and the total time spent in both the open and closed arms were recorded. In addition, the Anxiety index was calculated for each rat using the following formula: *Anxiety Index* = *1 − {[(Time in Open Arms/Total Time in all Arms)* + *(Open Arm Entries/Total Entries in all Arms)] ÷ 2}.* Higher anxiety index values reflect a behavioral pattern characterized by reduced exploration of the open arms, indicative of increased anxiety-like responses.

#### Assessment of spontaneous locomotor activity

The spontaneous locomotor activities of rats were recorded using the Ugo Basile Activity Cage (Italy) (Can et al., 2012).  Horizontal and vertical locomotor activities were monitored for 2 min, a duration deliberately selected to minimize novelty-induced stress and prevent habituation effects, particularly relevant when testing aged animals. Although some recent studies have used longer observation periods (e.g., 5–10 min), this shorter interval is sufficient to capture initial locomotor reactivity, and has been used consistently in our previous research (Tamba et al., 2009), ensuring methodological continuity and facilitating comparability of outcomes.

#### Evaluation of motor coordination using RotaRod test

The RotaRod device (Ugo Basile, Italy) was used to evaluate the therapeutic effects of the compound on motor performance in young adult and naturally aged rats, as described by Brooks et al. (2012). According to the protocol, the animals were not trained prior to testing, as we chose to use an accelerated speed protocol for motor coordination assessment. On the test day, rats were placed on the rotating rod, which accelerated from 2 to 20 rpm over a 5-min period. The latency to fall was recorded as an indicator of motor performance. For each rat, the rotarod test was performed in triplicate, with 40–45 min of rest between trials and the latency to fall and the rotation speed at fall were recorded for each trial. To provide an integrated measure of motor performance over time, area under the curve (AUC) values were calculated based on these repeated measures.

#### Sample collection and hematological analysis

Hematology assays: the selected animals were bled via jugular vein puncture under isoflurane anesthesia. Blood samples were collected into anticoagulant tubes containing 50 µl of 2% EDTA. Whole blood was used to measure hematological parameters including white blood cell count (WBC), red blood cell count (RBC), hemoglobin (Hb), hematocrit (HCT), mean corpuscular volume (MCV), mean corpuscular hemoglobin (MCH), mean corpuscular hemoglobin concentration (MCHC), platelet count (PLT), red cell distribution width (RDW), mean platelet volume (MPV), and differential WBC count (neutrophils, lymphocytes, eosinophils, monocytes, basophils). Analyses were conducted using the HEMAVET 950 automated blood cell analyzer (Drew Scientific Ltd). Reference ranges specific to line, sex, and age obtained in our laboratory were compared with those published by He et al., (2017).

#### Pro- and anti-inflammatory cytokines evaluation

To evaluate serum pro-inflammatory cytokines associated with aging, we used the LEGENDplex™ Rat Inflammation Panel V02 (BioLegend, CA, USA), a bead-based immunoassay designed for simultaneous quantification of multiple cytokines. The assay employs fluorescence-encoded beads coated with capture antibodies specific to each target cytokine: tumor necrosis factor-alpha (TNF-α), interleukin-10 (IL-10), interleukin-18 (IL-18), interleukin-1 beta (IL-1β), and interleukin-6 (IL-6). Data acquisition was performed using the NovoCyte 3005 Flow Cytometry System (Agilent, CA, USA). Cytokine concentrations were calculated using the LEGENDplex™ Data Analysis Software, based on standard curves generated from known concentrations of recombinant cytokines, following the manufacturer's instructions.

#### Histological assessment

At the end of the testing period, rats were euthanized via an overdose of isoflurane anesthesia. Subsequently, key organs—brain tissues, liver, kidneys, heart, and lungs—were harvested and prepared for analysis using histological and IHC techniques. IHC staining was performed in accordance with previously published protocols (Magaki et al. 2019; Guerin et al., 2023; Stanciu et al. 2024), employing antibodies detailed in the accompanying Table 1. The organs were further trimmed, fixed in 10% formaldehyde (Formaldehyde 36%, VWR Chemicals, Radnor, Pennsylvania, USA) for 24–48 h and processed using the Excelsior™ AS Tissue Processor (Epredia Holdings Ltd., Portsmouth, NH, USA), employing a standardized protocol comprising three sequential phases: graded dehydration (Ethanol 99,9%, Laboratorium Life Science, Galati, Romania) and clearing (Xylene, Epredia Holdings Ltd., Portsmouth, NH, USA), and paraffin wax infiltration (Histoplast PE, Epredia Holdings Ltd., Portsmouth, NH, USA), followed by embedding in paraffin wax blocks (Histoplast PE, Epredia Holdings Ltd., Portsmouth, NH, USA). Thin sections, 3 µm thick, were cut from the embedded tissue blocks using a semi-automatic microtome CUT 5062 (SLEE medical GmbH, Nieder-Olm, Germany), stained with hematoxylin (Mayer’s hemalum solution, Sigma-Aldrich, Burlington, USA) and eosin (Eosin Y-solution 0,5% aqueous, Sigma-Aldrich, Burlington, USA) (H&E), and examined under a Leica DM750 light microscope (Leica Microsystems GmbH, Wetzlar, Germany) (Kittel et al. 2004; Morawietz et al., 2004; Ruehl-Fehlert et al. 2003) at 400 × magnification scale. Photomicrographs were then analyzed and compared to the control by a veterinary histopathologist.

#### Immunohistochemical analysis based on histological findings

Immunohistochemical staining was performed using antibodies against microglial activation marker (CD68), interleukin-1 beta (IL-1β), tumor necrosis factor-alpha (TNF-α), proliferating cell nuclear antigen (PCNA), glial fibrillary acidic protein (GFAP), and cyclooxygenase-2 (COX-2). Tissue sections were dewaxed in xylene, followed by rehydration through a series of ethanol washes. Antigen retrieval was performed by microwaving the sections for 10 min at 95 °C in 10 mM citrate buffer (pH 6). After cooling for 20 min, the sections were washed twice with PBS for 5 min each. Endogenous peroxidase activity was blocked using 3% hydrogen peroxide, and the sections were rinsed with PBS. Primary antibodies were applied and incubated overnight at 4 °C in a humidified chamber (dilutions are detailed in Table 2). The following day, the slides were washed three times with PBS and incubated with secondary antibodies from the Leica Novocastra kit. Finally, the slides were developed using 3,3'-diaminobenzidine (DAB Substrate Kit AB64238) and counterstained with hematoxylin.

**Table 2:** Primary and secondary antibodies, along with their specific dilutions, employed in immunohistochemical analysis

Primary Antibody	Dilution	Secondary Antibody	Dilution
GFAP (SYSY Cat. No 173002), polyclonal rabbit antibody, SYSY Antibodies (Göttingen, Germany)	1:500	Goat anti-rabbit IgG H&L (HRP) **(**ab205718) Abcam (Cambridge, UK)	1:500
Anti-CD68 antibody [SP251], (ab192847), monoclonal rabbit antibody, Abcam (Cambridge, UK)	1:100	Goat anti-rabbit IgG H&L (HRP) Abcam** (**ab205718) (Cambridge, UK)	1:100
Invitrogen COX2 monoclonal mouse antibody (COX 229), Thermo Fisher Scientific (Waltham, MA, USA)	1:100	Goat anti-mouse IgG H&L (HRP) (ab97023) Abcam (Cambridge, UK)	1:100
Anti-rat TNF Alpha antibody (AAR33), Bio-Rad Antibodies (Kidlington, UK)	1:250	Goat anti-rabbit IgG H&L (HRP) (ab6721) Abcam (Cambridge, UK)	1:250
Rabbit anti-rat interleukin-1 beta (AAR15G) Bio-Rad Antibodies (Kidlington, UK)	1:250	Goat anti-rabbit IgG H&L (HRP) (ab6721) Abcam (Cambridge, UK)	1:250
Invitrogen PCNA polyclonal rabbit antibody (PAS-27214) Thermo Fisher Scientific (Waltham, MA, USA)	1:250	Goat anti-rabbit IgG H&L (HRP) (ab6721) Abcam (Cambridge, UK)	1:250
Anti-Caspase-3 rabbit antibody (A13916) (Cambridge, UK)	1:100	Goat anti-rabbit IgG H&L (HRP) **(**ab205718) Abcam (Cambridge, UK)	1:100

*GFAP* glial fibrillary acidic protein, *COX-2* cyclooxygenase 2, *TNF-α* tumour necrosis factor-alpha, *PCNA* Proliferating cell nuclear antigen, *HRP* horseradish peroxidase, *IgG* immunoglobulin G

All IHC staining was analyzed using the open-access QuPath software (v0.5.1) on SVS file format images (7.5 GB). The analysis workflow in QuPath included the following key steps: tissue detection, stain vector estimation, manual annotation of regions of interest (ROIs) using QuPath tools, cell detection, parameter optimization (including threshold 0.1 and sigma 1.5), positive cell detection, cell classification, and exporting measurements (Bankhead, et al., 2017).

Immunohistochemical findings were assessed both qualitatively and semi-quantitatively across multiple tissue sections from each experimental group. Representative images were selected based on prior histological evaluation to illustrate typical staining patterns. These selections reflect the exploratory nature of the study and are intended to provide preliminary insights that may support the rationale for future large-scale preclinical investigations. To investigate microglial activity, three distinct regions of the hippocampus (dentate gyrus (DG), CA-2, and CA-3) were selected based on their roles in neuroinflammation and microglial activation. The dentate gyrus is involved in neurogenesis, while the CA-2 and CA-3 regions are critical for synaptic plasticity and are often implicated in various neurodegenerative conditions. The analyzed parameters included positive cell density and positive cell count per area interest.

### Statistical analysis

All statistical analyses were conducted using R (R Core Team, 2020). The normality of each dataset was assessed using the Shapiro–Wilk test, and the homogeneity of variances was verified with Levene’s test. For normally distributed data with homogeneous variances, a one-way ANOVA was performed, followed by pairwise comparisons of estimated marginal means (EMMs) using either the Šídák or Bonferroni correction for multiple testing. For non-normally distributed data, the Kruskal–Wallis rank sum test was employed, followed by pairwise Wilcoxon rank sum tests (for independent samples) or Wilcoxon signed-rank tests (for paired samples), both using continuity correction. The Friedman rank sum test was used for analyzing repeated measures, such as body weight evolution over time, with post hoc analysis performed using pairwise Wilcoxon signed-rank tests corrected by the Bonferroni method. All *p*-values from post hoc analyses were corrected for multiple comparisons as appropriate. Statistical significance thresholds were defined as: *p* < 0.05 (*), *p* < 0.01 (**), and *p* < 0.001 (***). Results are reported as group mean ± standard error of the mean (SEM), unless otherwise specified. All graphical representations, including bar plots and line graphs, with error bars, were generated using *ggplot2* (Wickham, 2016)  in R. Where applicable, global test results (e.g., ANOVA, Friedman) and adjusted *p*-values from post hoc comparisons were displayed directly on the figures using *ggpubr* functions for enhanced clarity and interpretability.

## Results

### General health parameters

**Fig. 2:**
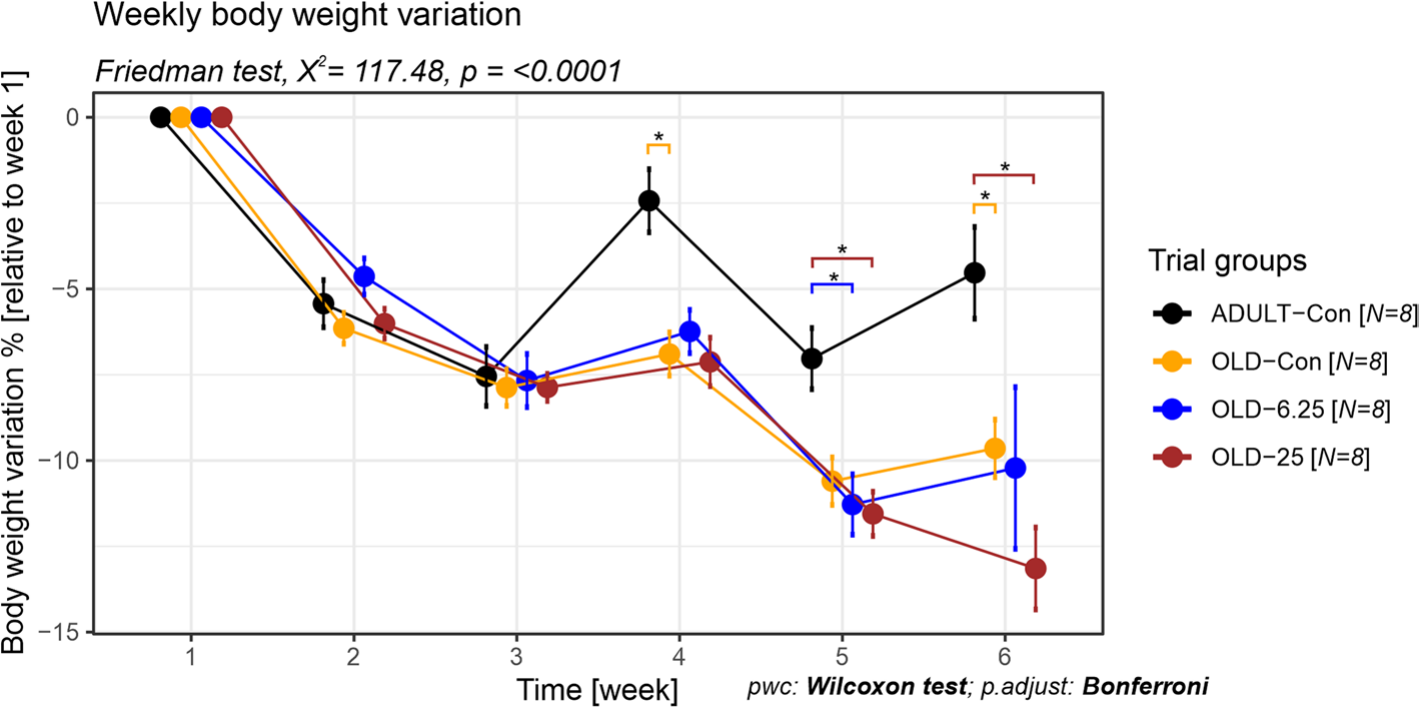
The impact of Cannabixir® Medium Flos on weight variation in old and adult rats. We evaluated the weight variation in rats over a 6-week period by weekly body weight measurements. Weight variation percentages of the animals are expressed as percentages of their initial weight (baseline weight recorded in week 1). Data are expressed as group mean ± standard error of the mean (SEM). Treatment group abbreviations: ADULT-Con: the young adult control group; OLD-Con: the naturally aged control group; OLD-6.25: the Cannabixir® Medium Flos 6.25 mg/kg naturally aged group; and OLD-25: the Cannabixir® Medium Flos 25 mg/kg naturally aged group. Significance codes: * *p* < 0.05; ** *p* < 0.01; *** *p* < 0.001

The impact of Cannabixir® Medium Flos (6.25 mg/kg and 25 mg/kg) on rats maintained on a standard diet was investigated by measuring the variation in individual percentage weight over time, relative to the initial body weight. Globally, over the entire experimental period, all groups experienced a decrease in body weight. However, the percentage of weight loss varied among groups, between 2.43% for the ADULT-Con in the fourth week and 13.15% for the OLD-25 groups in the last week. Our analysis revealed no significant weekly weight differences among the old animal groups, regardless of the administered dose. In contrast, significant differences were observed between adult and old rats, with or without cannabinoid treatment (*p* < 0.0001, Friedman test, χ^2^ = 117.47, df = 5). Specifically, at week 4, the ADULT-Con group exhibited a lower weight loss (− 2.43%) compared to the OLD-Con group (− 6.90%), representing a 4.47% smaller decrease (*p* = 0.047, Wilcoxon rank sum test, p adjusts Bonferroni). This trend continued in week 6, with the ADULT-Con group showing a − 4.53% weight change versus − 9.65% in the OLD-Con group (a 5.12% difference, *p* = 0.047). Furthermore, in week 5, the ADULT-Con group showed a − 7.03% change, while the OLD-25 and OLD-6.25 groups had higher losses of − 11.55% and − 11.28%, respectively—corresponding to differences of 4.52% and 4.25% (both *p* = 0.047). Additionally, in week 6, the ADULT-Con group maintained a significantly lower weight loss compared to OLD-25 (− 13.15%), with an 8.62% difference (*p* = 0.047), (Fig. 2).

Oral administration of Cannabixir® Medium Flos in aged rats led to significant changes in leukocyte distribution, specifically affecting neutrophil and lymphocyte percentages. A one-way ANOVA revealed a significant treatment effect on neutrophil levels (F(3,24) = 4.46, *p* = 0.0126), with a moderate to large effect size (partial η^2^ = 0.36, 95% CI [0.063–1.000]). Post-hoc comparisons (Šídák-adjusted) indicated that the 25 mg/kg group (OLD-25) had significantly higher neutrophil percentages compared to both the naturally aged control group (OLD-Con, *p* = 0.0498) and the adult control group (ADULT-Con, *p* = 0.0241). Similarly, a significant treatment effect was observed for lymphocyte percentages (F(3,24) = 4.41, *p* = 0.0132), also with a moderate to large effect size (partial η^2^ = 0.36, 95% CI [0.060–1.000]). Notably, the OLD-25 group displayed a significantly lower lymphocyte proportion compared to the ADULT-Con group (*p* = 0.0187). No significant differences were observed between the OLD-6.25 and OLD-Con groups for either neutrophil or lymphocyte values. In addition to leukocyte changes, one-way ANOVA revealed a significant treatment effect on mean corpuscular volume (MCV) across groups (F(3,24) = 6.82, *p* = 0.0018), with a large effect size (partial η^2^ = 0.46, 95% CI [0.165–1.000]). Post-hoc comparisons (Šídák-adjusted) showed that the 6.25 mg/kg group (OLD-6.25) had significantly lower MCV values compared to both the OLD-Con group (*p* = 0.0298) and the OLD-25 group (*p* = 0.0113). Furthermore, the OLD-25 group exhibited significantly higher MCV compared to the ADULT-Con group (*p* = 0.0191), while no significant difference was found between the OLD-Con and OLD-25 groups (*p* = 0.9991). Importantly, all MCV values remained within the established physiological reference ranges for Sprague–Dawley rats.

### Effects of Cannabixir® Medium Flos, a cannabinoid receptor ligand, on locomotion and anxiety-related responses

Chronic, intermittent oral administration of Cannabixir® Medium Flos at doses of 6.25 mg/kg and 25 mg/kg in aged rats did not significantly impact overall motor coordination or endurance capacity. The RotaRod performance, as measured by latency to fall and speed (the performance test was repeated three times and the results were used to calculate the area under the curve—AUC), showed no significant differences among the groups (latency to fall: F(3,24) = 0.32, *p* = 0.8139, partial η^2^ = 0.038, 95% CI [0–1]; speed when fall occurs: F(3,24) = 0.38, *p* = 0.7707, partial η^2^ = 0.045, 95% CI [0–1]). Post-hoc comparisons using Šídák-adjusted *p*-values confirmed the absence of statistically significant differences between the OLD-Con and ADULT-Con groups (latency to fall: *p* = 0.9993; speed when fall occurs: *p* > speed when fall occurs: *p* = 0.9992), as well as between any treated (OLD-6.25 & OLD-25 a) and control group (OLD-Con, latency to fall: *p* > 0.9287; speed when fall occurs: *p* > 0.8951) (Fig. 4A and 4B).

Similarly, the Treadmill Fatigue Test, which evaluated physical performance through work performed (joule) and power output (watts), revealed no statistically significant treatment effects (work: F(3,24) = 1.88, *p* = 0.1597, partial η^2^ = 0.19, 95% CI [0–1]; power: F(3,24) = 0.97, *p* = 0.4241, partial η^2^ = 0.10). Post-hoc comparisons showed no significant differences among all group contrasts, including between the OLD-25 and OLD-Con groups (*p* = 0.6745) and the OLD-6.25 and ADULT-Con groups (*p* = 1.0000) (Fig. 4C and 4D).

Assessment of spontaneous locomotor behavior in the Activity Cage, measured by horizontal and vertical movement counts, revealed no significant changes in exploratory activity or cannabinoid-induced sedation. Although a numerical decrease in horizontal activity (F(3,24) = 2.46, *p* = 0.0872, partial η^2^ = 0.24, 95% CI [0–1]) was observed in OLD-6.25 (418 movements) and OLD-25 (357 movements) compared to OLD-Con (465 movements), none of these differences reached statistical significance (*p* > 0.1052), suggesting no relevant suppression of locomotor function (Fig. 4E). In contrast, vertical movement counts showed more variability between groups. While the one-way ANOVA did not indicate a statistically significant treatment effect (F(3,24) = 1.83, *p* = 0.1681), the partial η^2^ value of 0.186 suggests a small-to-moderate effect size. The OLD-Con group (65 movements) exhibited a noticeable decrease in vertical movements compared to the ADULT-Con group (114 movements, *p* = 0.3045), while the OLD-6.25 group demonstrated rearing activity nearly restored to adult levels (110 movements, *p* = 0.3869). The OLD-25 group (81 movements), exhibited a wider range of responses and greater variability (standard deviation = 69.14), suggesting heterogeneous behavioral sensitivity at the higher dose. Although none of these differences reached statistical significance in post-hoc comparisons (Šídák-adjusted *p* > 0.30), the vertical activity profile highlights potential dose- and age-related trends in cannabinoid response (Fig. 4F).

Anxiety-like behavior was evaluated using the Elevated Plus Maze (EPM), with outcomes measured as the number of entries and time spent in both the open and closed arms. Between-group comparisons showed no significant treatment effect for either parameter (open arm entries: *Kruskal–Wallis, χ*^*2*^*(3)* = *1.3, p* = *0.73*; closed arm entries: *ANOVA, F(3,25)* = *0.28, p* = *0.84,* ${\eta }_{g}^{2}$= *0.03*; time spent in open arms: *Kruskal–Wallis, χ*^*2*^*(3)* = *2.33, p* = *0.51*; time spent in closed arms: *ANOVA, F(3,25)* = *0.54, p* = *0.66,* ${\eta }_{g}^{2}$= *0.06*). The omnibus tests (One-way ANOVA or Kruskal–Wallis), selected based on the normality assumption, indicated similar performance across groups (Fig. 5A -D), and post-hoc comparisons using Bonferroni correction confirmed the absence of significant differences between the adult control group (ADULT-Con, *p* = 1.0) and any of the aged groups, including the Cannabixir®-treated animals (OLD-Con, OLD-6.25 and OLD-25, *p* = 1.0).

In addition, anxiety indices—calculated based on open arm exploration and entry ratios—were analyzed to further evaluate anxiety-like behavior. The OLD-6.25 and OLD-25 groups, which received chronic intermittent oral administration of Cannabixir® Medium Flos, exhibited numerically lower anxiety indices (0.834 and 0.824) compared to both control groups, young adults (ADULT-Con, 0.856) and naturally aged individuals (OLD-Con, 0.903), suggesting a potential reduction in anxiety-like responses. However, these differences did not reach statistical significance (Kruskal–Wallis χ^2^(3) = 1.48, *p* = 0.69), and no significant pairwise comparisons (*p* = 1.0) were observed after Bonferroni adjustment (Fig. 5E).

### The impact of pharmacological modulation of the endocannabinoid system with Cannabixir® Medium Flos on age-related cell proliferation, neuroinflammation and glial reactivity

Histopathological examination using hematoxylin–eosin (H&E) staining of major organs (liver, kidneys, heart, and lungs) revealed no significant alterations in the treatment groups compared with controls (Fig. 6). In brain sections from ADULT-Con rats, H&E staining revealed the dentate gyrus as a prominent, darkly stained V-shaped structure, with its open arm enclosing the *cornu ammonis* 3 (CA3) region of the hippocampus. On both sides of CA3, the dentate gyrus displayed three well-defined layers extending toward the hilus. The suprapyramidal (inner, S) and infrapyramidal (outer, I) blades were clearly identifiable, merging at the crest (C), where both blades converge (also shown in Fig. 6). Each blade was composed of three histologically distinct layers: the molecular layer (M), the granule cell layer (G), and the polymorphic layer (P). The molecular layer appeared continuous with that of the adjacent hippocampal formation within the depths of the hippocampal fissure. The granule cell layer consisted of 5 to 7 rows of tightly packed, uniform granule cells, with spherical to oval morphology and rounded vesicular nuclei. Beneath it, the polymorphic layer contained axons of granule cells, dendritic processes from CA3 pyramidal neurons, and numerous small, densely stained interneurons. Additionally, a small number of large, pyramidal-like basket cells with vesicular nuclei were observed across all three layers (Fig. 6). However, in the OLD-6.25 group, the granule cell layer (G) of the dentate gyrus exhibited 4–5 rows of densely packed spherical or oval cells with rounded vesicular nuclei. Additionally, in the subgranular zone (SGZ) and at the crest of the dentate gyrus, cells with occasional hyperchromatic nuclei and scattered elongated cells with dense nuclei were observed.

Similar aspects were observed in the granular layer of the dentate gyrus in the OLD-25 group, which exhibited 3–5 rows of densely packed cells with euchromatin-rich nuclei and visible nucleoli in both the suprapyramidal (S) and infrapyramidal (I) zones. In the subgranular zone (SGZ), 2–3 rows of cells with dense nuclei were present, particularly at the crest of the dentate gyrus. These findings are comparable to those observed in the ADULT-Con rats, in which the dentate gyrus displayed 5–7 rows of densely packed cells with euchromatin-rich nuclei and prominent nucleoli in the granular layer. In contrast, the OLD-Con group showed a marked reduction in the number of nucleated cells within the granular layer, along with an increased presence of elongated cells with hyperchromatic nuclei in the SGZ.

Furthermore, to investigate the effects of prolonged drug therapy on molecular markers related with age-associated cognitive decline and the endocannabinoid system, the study evaluated: (1) cell proliferation and apoptosis through PCNA and caspase-3, which may provide indirect insights into neurogenic activity; (2) neuroinflammatory processes via TNF-α, IL-1β, and COX-2; and (3) glial activation by assessing CD68 and GFAP expression.

Considering the dual function of glial cells in the brain, particularly in the context of aging—where they maintain homeostasis and mitigate neurotoxicity through degradation and phagocytosis, but also promote neuroinflammation by releasing inflammatory cytokines—the effect of chronic cannabinoid therapy on astrogliosis was evaluated (Quincozes-Santos et al. 2021). Using GFAP to quantify reactive astrocytes, we observed an increased number of small to medium-sized astrocytes with darkly stained extensions in the DG and Ammon’s horn. Conversely, astrocytes in the OLD-Con group frequently appeared as cellular debris or intensely stained with variable sizes.

There was a significant increase in GFAP-positive astrocyte immunolabeling in the OLD-25 group compared to both the OLD-Con group (*p* = 0.03) and the ADULT-Con group (*p* = 0.03). No significant differences were found between the ADULT-Con and OLD-Con groups (*p* = 0.27), between the OLD-Con and OLD-6.25 groups (*p* = 0.06), or between the OLD-6.25 and OLD-25 groups (*p* = 0.23). The highest average number of GFAP-immunolabeled cells was observed in the OLD-25 group (535.50 ± 45.96), and the lowest in the OLD-Con group (180.50 ± 50.50) (Fig. 7). Regarding CD68 staining, no significant differences were observed between the ADULT-Con and OLD-6.25 groups (*p* = 0.83), or between the ADULT-Con and OLD-25 groups (*p* = 0.74). The OLD-Con group exhibited the highest number of CD68-immunolabeled macrophages, with an average of 558.50 ± 58.69 positive cells, significantly higher than in the other groups (*p* < 0.05). CD68-labeled macrophages displayed an elongated morphology with intense nuclear and cytoplasmic staining (Fig. 7).

In ADULT-Con and OLD-6.25 rats, PCNA-positive cell nuclei appeared as perivascular agglomerates (Fig. 8). In OLD-25 rats, PCNA-positive nuclei were diffusely distributed. Caspase-3 positive cells remained sparse around neurons in the CA1 region, and the number of neuronal rows showed no significant variations. In contrast, in OLD-Con rats, clusters of PCNA-positive cells were observed around blood vessels near the dentate gyrus (DG) region. Additionally, caspase-3 expression in the DG region was characterized by a reduction to 1–2 rows of granular cells with hyperchromatic, elongated nuclei (Figs. 7 and 8). In OLD-Con rats, the average number of PCNA-positive nuclei observed as perivascular clusters was 15.50 ± 3.54, significantly lower than in the other groups (*p* < 0.05). The highest PCNA nuclear labeling was observed in the ADULT-Con group (141.50 ± 14.85), significantly higher than in the OLD-Con group (*p* = 0.05), followed by the OLD-6.25 group (129.50 ± 10.61), which was significantly higher than in the OLD-25 group (*p* = 0.05) (Fig. 8).

Immunolabeling for neuroinflammatory markers showed a significantly higher mean number of positive cells in the OLD-Con group: 795 ± 42.43 IL-1β-positive cells, 818 ± 16.97 TNF-α-positive cells, and 561 ± 14.14 COX-2-positive cells. These were significantly higher compared to the ADULT-Con, OLD-6.25, and OLD-25 groups (*p* < 0.01). Specifically, exposure of aged rats to 6.25 mg/kg cannabis resulted in the lowest mean number of cells positive for neuroinflammatory markers, with significantly fewer IL-1β and TNF-α-positive cells in the OLD-6.25 group compared to the OLD-25 group (*p* < 0.05). In the granular layer of the dentate gyrus (DG) region, caspase-3 positive cells were rarely detected among or surrounding the neurons. In the OLD-Con group (204.50 ± 30.40), the mean number of PCNA-labeled cells was significantly higher compared to the ADULT-Con group (*P* < 0.01), as well as compared to the OLD-25 group (*P* < 0.01) and the OLD-6.25 group (*P* < 0.05). No significant differences were found between the ADULT-Con, OLD-6.25, and OLD-25 groups. Additionally, caspase-3 expression in the granular layer of the DG region was characterized by a reduction to 1–2 rows of granular cells with hyperchromatic, elongated nuclei.

In both ADULT-Con and OLD-6.25 animals, the dentate gyrus (DG) exhibited 4–6 layers of cells in the granular layer (S and I zones), characterized by large nuclei with prominent nucleoli and abundant euchromatin. Occasional cells with elongated, hyperchromatic nuclei were observed in the subgranular zone (SGZ) of the DG, which were positively stained for IL-1β, TNF-α, and COX-2 (Figs. 7 and 8). In OLD-25 rats, treated with the highest dose, zonal changes were observed. The granular layer of the DG contained 3–5 layers of cells with large nuclei and prominent nucleoli, transitioning to elongated nuclei rich in heterochromatin towards the SGZ layer. In contrast, the OLD-Con group showed a reduction in the number of cells in the granular layer of the DG, with only 1–3 layers of cells, whose nuclei were rich in euchromatin. In the SGZ, the cells predominantly featured hyperchromatic, elongated nuclei and were positive for the pro-inflammatory markers IL-1β, TNF-α, and COX-2 (Figs. 7 and 8).

To assess systemic inflammation, the plasma concentrations of key pro- and anti-inflammatory cytokines (TNF-α, IL-1β, IL-6, IL-10, and IL-18) were measured following chronic intermittent oral administration of Cannabixir® Medium Flos. Among these, only TNF-α levels showed a statistically significant treatment-related effect (Fig. 9), as revealed by the Kruskal–Wallis rank sum test (χ^2^ = 16.25, *p* = 0.0006). Post-hoc comparisons using Bonferroni-adjusted Wilcoxon tests indicated that TNF-α was significantly elevated in the OLD-6.25 group compared to the OLD-Con group (*p* = 0.0018). No significant differences were found between the OLD-25 group and any other group (*p* > 0.26). This suggests a dose-dependent modulation of TNF-α, particularly at the 6.25 mg/kg dose. For all other cytokines—IL-1β, IL-6, IL-10, and IL-18—no statistically significant treatment effects were detected (all Kruskal–Wallis *p* > 0.28). IL-1β and IL-6 exhibited high variability within the aged groups, but these did not translate into statistically meaningful differences. Additionally, IL-10 and IL-18 levels remained low or undetectable in most samples, especially in the ADULT-Con group, with no significant pairwise group differences observed (*p* = 1.000 across comparisons). Collectively, these findings indicate that Cannabixir® Medium Flos does not broadly alter systemic cytokine balance, though it may selectively increase TNF-α levels at lower doses, possibly reflecting an age- or dose-specific immune modulation effect.

## Discussion

As research into the ECS advances, its therapeutic potential becomes increasingly apparent. Aging—a primary risk factor for cognitive decline, involves neuroanatomical and neurochemical changes that are mirrored by alterations in the ECS (Legdeur et al. 2018). Recent evidence suggest that interactions between the aging brain and the ECS (Hazzaa, et al., 2021), particularly through its modulatory receptors, could influence brain functions related to motor skills, emotions and cognition (Fogaca et al., 2018; Ruiz de Martín et al., 2022). However, targeting the ECS presents challenges, as CB1 and CB2 receptors play crucial roles in regulating neuronal function, leading to diverse effects on neuronal responses (Kamaruzzaman et al. 2023).

**Fig. 3:**
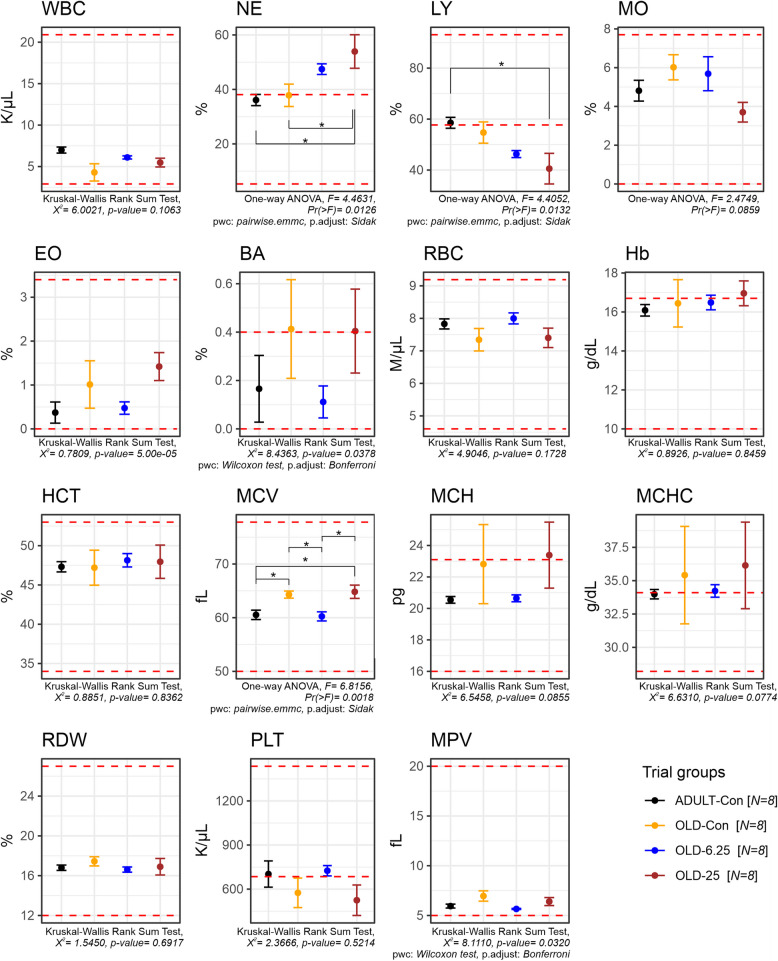
Evaluation of hematological parameters in rat blood samples after chronic intermittent administration of cannabinoids treatment. The red-dotted line highlights reference range for normal values. Data are expressed as group mean ± standard error of the mean (SEM). Parameter abbreviations: WBC: white blood cell count(k/µL); EO: eosinophils (%); NE: neutrophils (%); BA: basophils (%); LY: lymphocyte ((%); MO: monocyte (%); RBC: red blood cell count (M/µL); Hb: hemoglobin (g/dL); HCT: hematocrit (%); MCV: mean corpuscular volume (fL); MCH: mean corpuscular hemoglobin (pg); MCHC: mean corpuscular hemoglobin concentration (g/dL); PLT: platelet count (k/µL); RDW: red cell distribution width (%); MPV: mean platelet volume and differential (fL). Treatment group abbreviations: ADULT-Con: the young adult control group; OLD-Con: the naturally aged control group; OLD-6.25: the Cannabixir® Medium Flos 6.25 mg/kg naturally aged group; and OLD-25: the Cannabixir® Medium Flos 25 mg/kg naturally aged group. Significance codes: * *p* < 0.05; ** *p* < 0.01; *** *p* < 0.001

Consistent with earlier preclinical and clinical studies (Stanciu et al. 2024; Alshaarawy and Anthony 2019), which reported little to no impact of Cannabis-active constituents on weight gain, our study also observed only a slight weight loss across all groups throughout the experimental period. There were no significant differences in weekly weight variation among the older animal groups, regardless of the Cannabixir® Medium Flos dose (6.25 mg/kg or 25 mg/kg), suggesting that chronic intermittent treatment did not markedly influence weight dynamics in aged rats (Fig. 2). However, statistically significant differences were observed between adult and aged rats across several time points, particularly between weeks 4 and 6 (e.g., 4.47% difference at week 4, *p* = 0.047, Fig. 2). These findings indicate that aging has a stronger impact on body weight regulation than cannabinoid treatment per se. The better weight maintenance observed in adult rats may reflect age-related declines in metabolic and physiological efficiency in older animals. Supporting the weight-related findings, the hematological evaluation indicated a generally safe profile of Cannabixir® Medium Flos therapy, with all measured parameters remaining within physiological reference ranges for Sprague–Dawley rats (Fig. 3). However, statistically significant hematological changes were observed in the OLD-25 group (25 mg/kg), including increased neutrophil percentages and decreased lymphocyte proportions, suggesting a mild alteration in immune cell distribution. These shifts may reflect subtle effects on erythropoiesis or immune responses, potentially linked to cannabinoid-mediated changes in metabolism or nutrient absorption. Additionally, significant changes in mean corpuscular volume (MCV) were detected, with OLD-6.25 rats showing lower values and OLD-25 rats higher values compared to controls, although still within normal limits (Fig. 3). These findings highlight the importance of further research to clarify the immunomodulatory effects of cannabinoid-based treatments in aged populations and to better understand their implications for long-term safety and pharmacotherapeutic use.

**Fig. 4:**
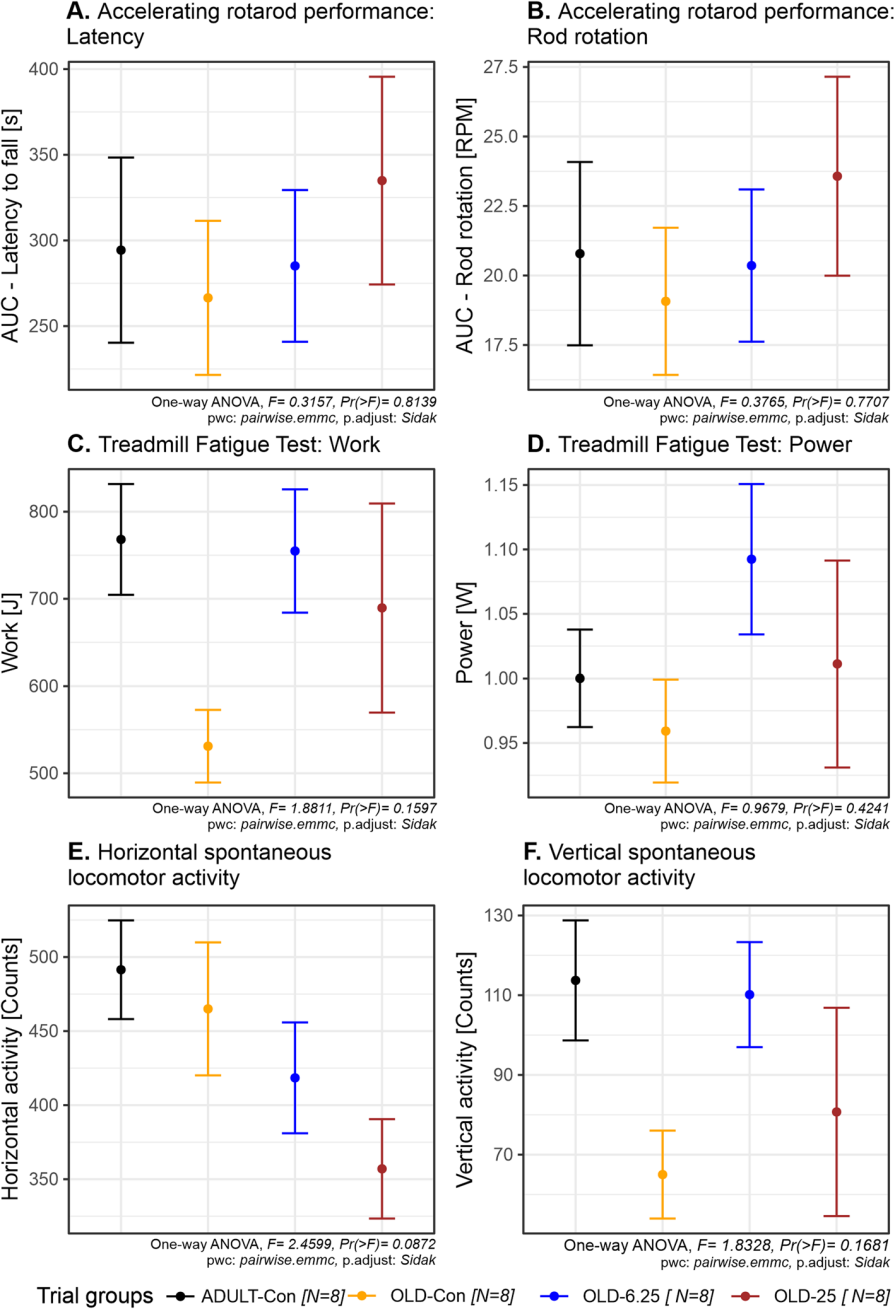
The effect of Cannabixir® Medium Flos on endurance, fatigue, and spontaneous locomotor activity in rats. **A**. Latency to fall in the accelerating speed test (the rotarod performance test was repeated three times and the results were used to calculate the area under the curve—AUC). **B**. Rotation speed when fall occurs in the accelerating speed test (the rotarod performance test was repeated three times and the results were used to calculate the area under the curve—AUC). **C**. Work performed during the treadmill fatigue tolerance test. **D**. Power output during the treadmill fatigue tolerance test. **E**. Horizontal spontaneous locomotor activity. **F**. Vertical spontaneous locomotor activity. Data are expressed as group mean ± standard error of the mean (SEM). Treatment group abbreviations: ADULT-Con: the young adult control group; OLD-Con: the naturally aged control group; OLD-6.25: the Cannabixir® Medium Flos 6.25 mg/kg naturally aged group; and OLD-25: the Cannabixir® Medium Flos 25 mg/kg naturally aged group. Significance codes: * *p* < 0.05; ** *p* < 0.01; *** *p* < 0.001

**Fig. 5:**
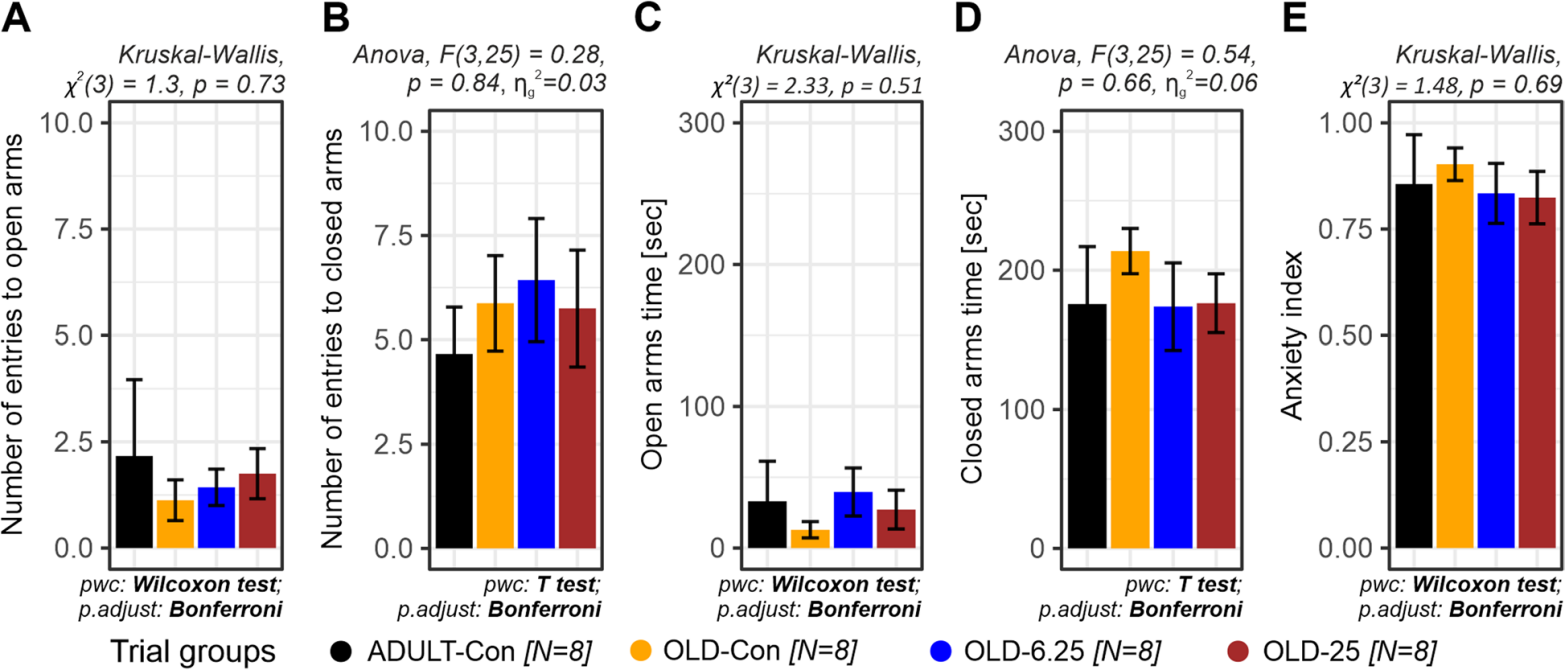
The anxiety-like behavior after chronic intermittent oral Cannabixir® Medium Flos exposure in rats. **A**. Number of entries into open arms. **B**. Number of entries into closed arms. **C**. Time spent in open arms (seconds). **D**. Time spent in closed arms (seconds). **E**. Anxiety index estimated based on open arm exploration and entry ratios. Data are expressed as group mean ± standard error of the mean (SEM). Treatment group abbreviations: ADULT-Con: the young adult control group; OLD-Con: the naturally aged control group; OLD-6.25: the Cannabixir® Medium Flos 6.25 mg/kg naturally aged group; and OLD-25: the Cannabixir® Medium Flos 25 mg/kg naturally aged group. Significance codes: * *p* < 0.05; ** *p* < 0.01; *** *p* < 0.001

Chronic, intermittent administration of Cannabixir® Medium Flos in both young adult and naturally aged rats did not result in statistically significant changes in motor coordination, endurance, exploratory behavior, or anxiety-like responses. Performance across all behavioral assays—including the RotaRod, Treadmill Fatigue Test, Activity Cage, and Elevated Plus Maze—performance remained comparable between treated and control groups (Figs. 4 and 5). These findings suggest that, at the tested doses, Cannabixir® Medium Flos does not impair locomotor activity or induce anxiety-related alterations, which supports its safety profile in aged models. While minor, non-significant trends, such as a slight reduction in spontaneous locomotion at higher doses, were observed, they did not reach statistical significance and should be interpreted with caution (Fig. 4E and 4 F). The consistency of these results across multiple behavioral paradigms aligns is consistent with previous studies reporting neutral (Varvel et al. 2001) or dose-dependent anxiolytic effects of cannabinoids, particularly those with low ∆9-THC content (Zuardi et al. 2012; Berger et al. 2022; Martin et al. 2021). Although low doses of ∆9-THC have often been associated with anxiolytic effects—potentially through the modulation of approach behavior and the inhibition of anxiety-related circuits involving the prefrontal cortex and amygdala (Rubino et al. 2007), recent evidence suggests that this is not a universal outcome (Ramis, et al., 2016). Specifically, studies have shown that ∆9-THC can produce anxiogenic effects even at low doses, particularly in female subjects, indicating that sex-related differences in cannabinoid sensitivity may critically influence behavioral responses (Salviato et al., 2021). These findings emphasize the biphasic and context-dependent nature of THC’s effects on anxiety, and the importance of considering sex as a biological variable when evaluating cannabinoid-induced emotional modulation (Sharpe et al. 2020). Overall, these data support the notion that Cannabixir® Medium Flos is behaviorally well-tolerated and reinforces its potential for further investigation as part of a neuroprotective strategy, particularly in aging contexts. Future studies should explore long-term administration, a broader dose range, and incorporate additional neurobehavioral endpoints to fully characterize its efficacy and mechanism of action.

**Fig. 6:**
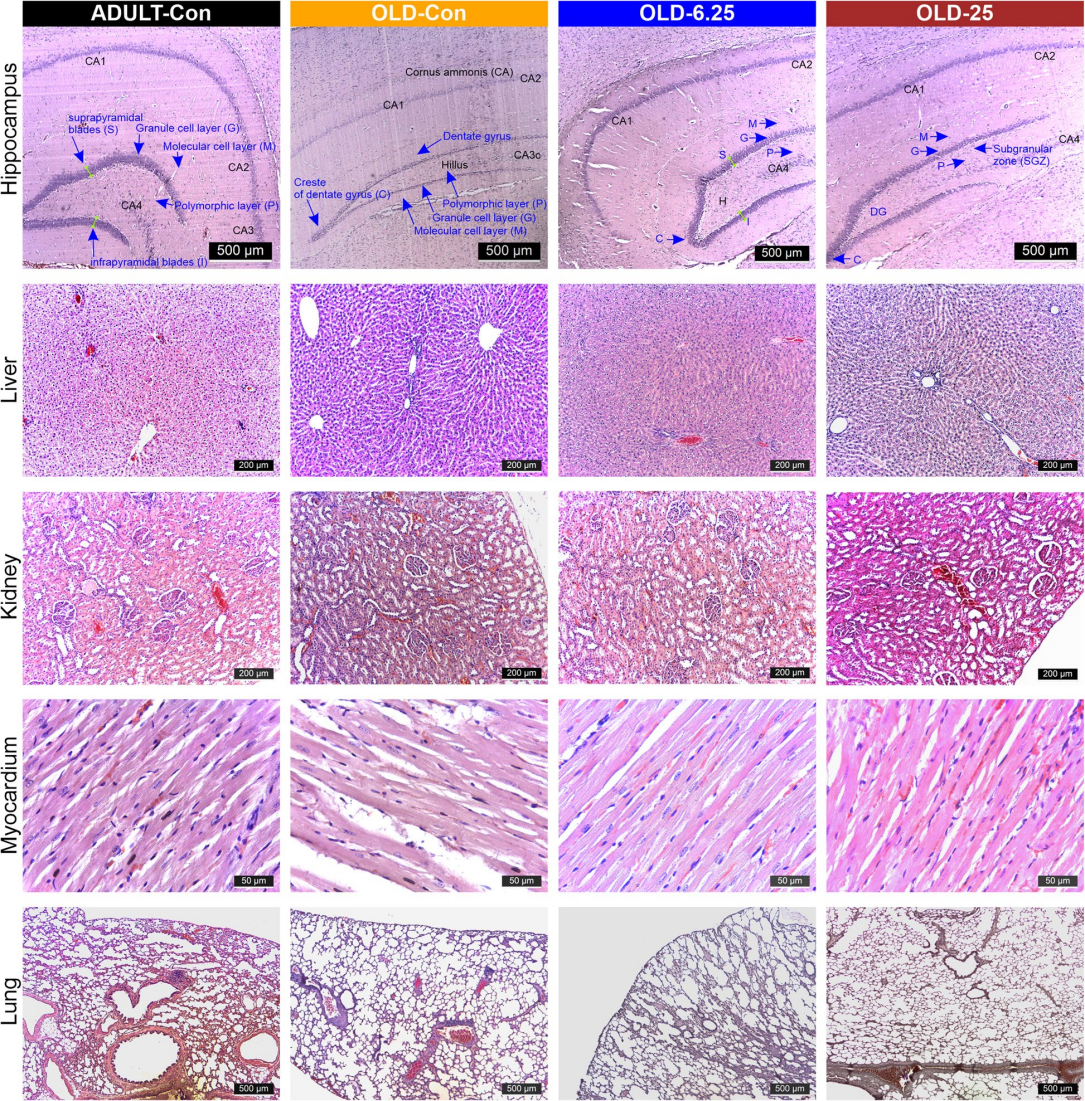
Histopathological assessment of the examined cannabinoids effects on selected internal organs. The chronic, intermittent administration of Cannabixir® Medium Flos did not result in significant changes in hematoxylin–eosin staining of key organs including lungs, myocardium, kidneys, and liver in the studied groups. Treatment group abbreviations: ADULT-Con: the young adult control group; OLD-Con: the naturally aged control group; OLD-6.25: the Cannabixir® Medium Flos 6.25 mg/kg naturally aged group; and OLD-25: the Cannabixir® Medium Flos 25 mg/kg naturally aged group; DG: dentate gyrus

**Fig. 7:**
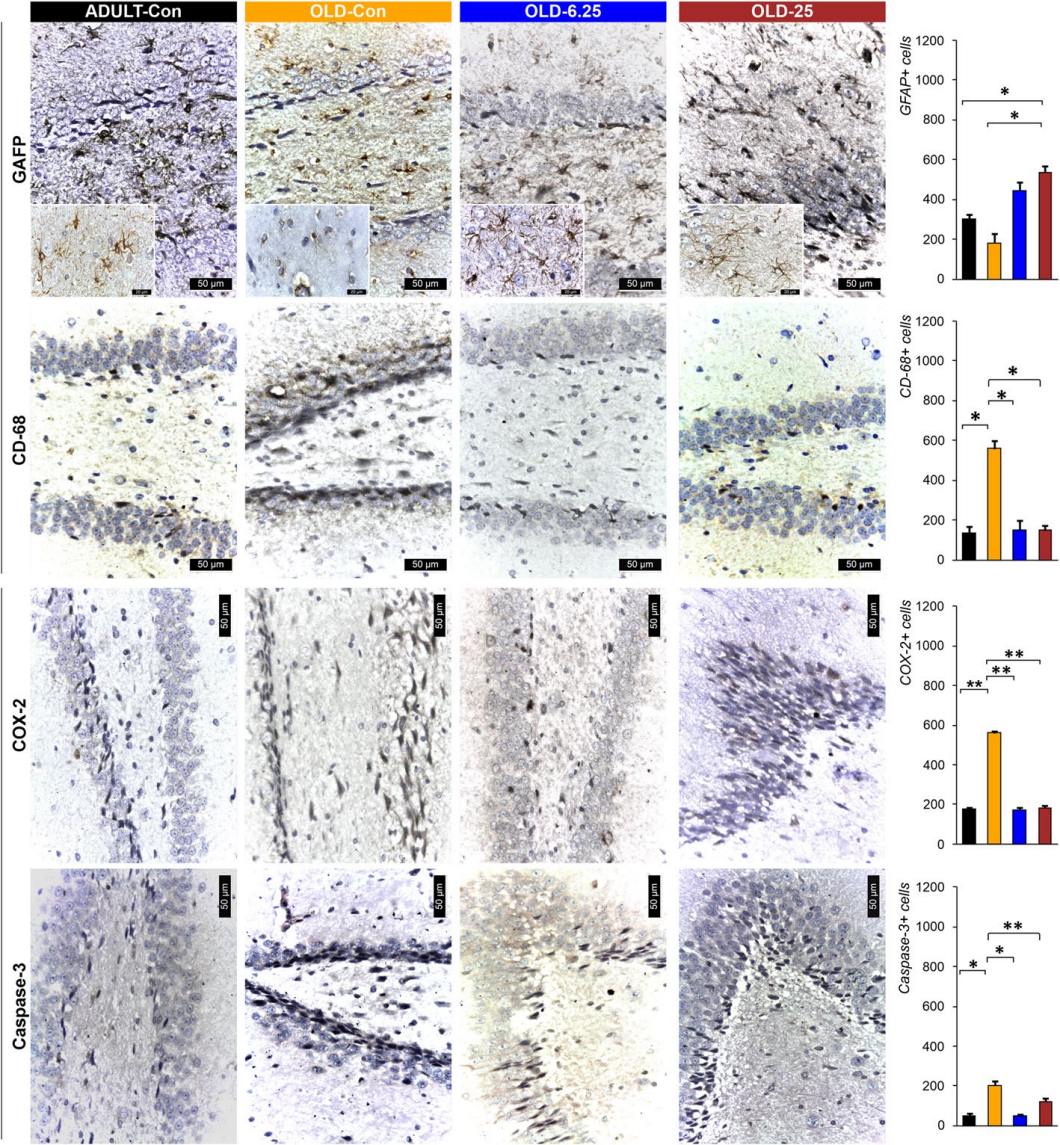
Representative immunohistochemical images show astrocytes and COX-2 expression in the DG and Ammon's horn (CA-1) regions. Quantification using GFAP and CD68 antibodies indicated an increase in small to medium-sized astrocytes with dark extensions in OLD-6.25 and OLD-Con, compared to ADULT-Con. In the OLD-Con group, astrocytes showed signs of degeneration, appearing as cellular debris or exhibiting markedly intense GFAP labeling. CD68-positive macrophages were predominantly observed in the OLD-Con group, where they presented elongated morphologies and strong nuclear and cytoplasmic staining, while fewer such cells were detected in the ADULT-Con, OLD-6.25, and OLD-25 groups. COX-2 immunoreactivity was relatively uniform across the ADULT-Con, OLD-6.25, and OLD-25 groups, with a notable increase observed in the OLD-Con group. ADULT-Con: the young adult control group; OLD-Con: the naturally aged control group; OLD-6.25: the Cannabixir® Medium Flos 6.25 mg/kg naturally aged group; and OLD-25: the Cannabixir® Medium Flos 25 mg/kg naturally aged group; GFAP: glial fibrillary acidic protein; COX-2: cyclooxygenase 2; CD68: microglial activation marker. Quantification of GFAP, CD68, and COX-2 positive cells in the hippocampal dentate gyrus across different groups. Bars represent the mean ± SEM. Statistical significance was assessed using the Student's t-test: * *p* < 0.05. Quantification of Caspase-3 positive cells in the hippocampal dentate gyrus across different groups. Bars represent the mean ± SEM. Statistical significance was assessed using the Student's t-test: * *p* < 0.05, ** *p* < 0.01

**Fig. 8:**
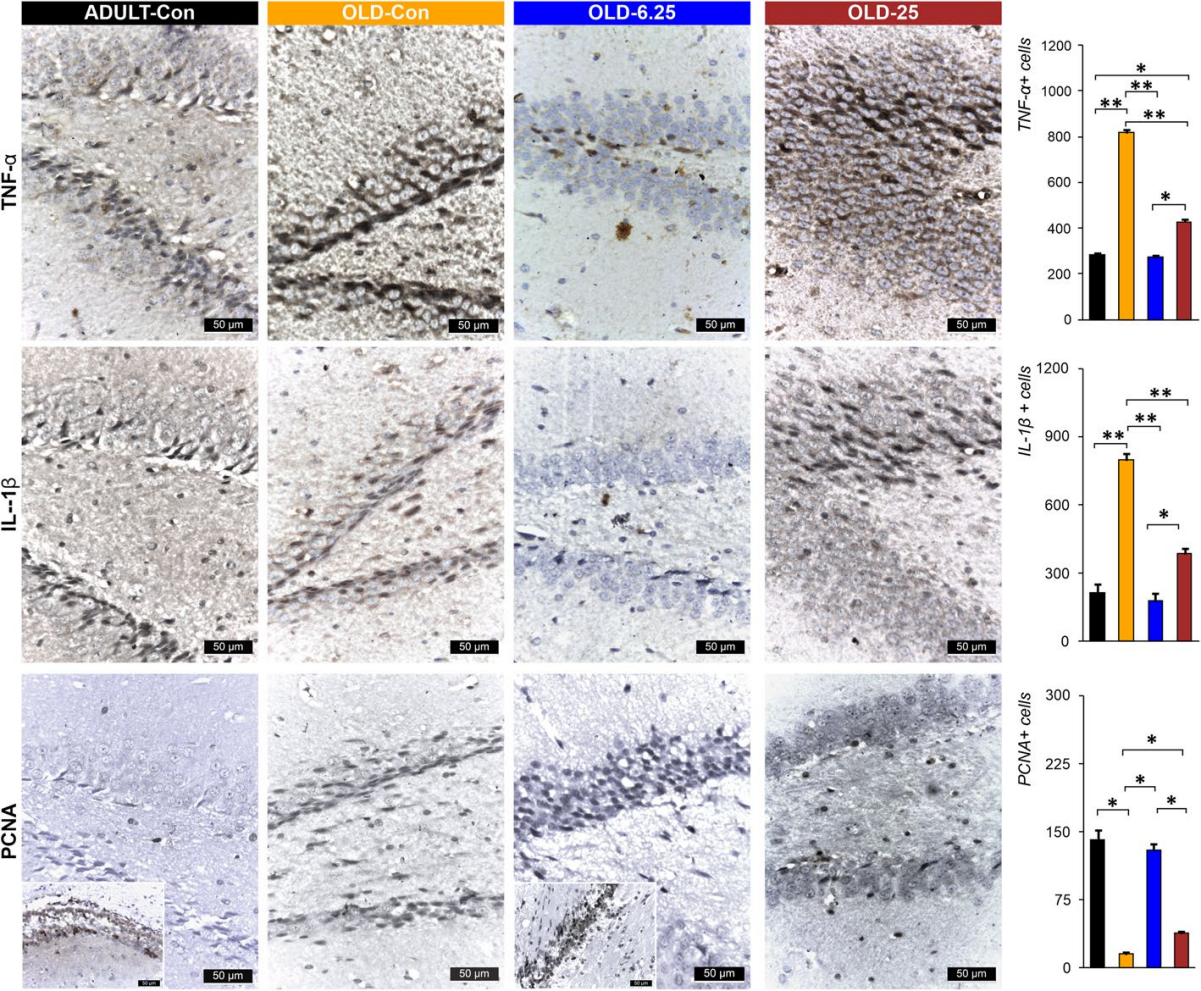
Representative images of the Cannabixir® therapy effect on the expression of PCNA, IL-1β and TNF-α. The results reveal heightened IL-1β and TNF-α levels alongside reduced PCNA expression in the hippocampal regions of the OLD-Con group, underscoring an amplified inflammatory response associated with aging. Conversely, the ADULT-Con and OLD-6.25 groups display lower IL-1β and TNF-α levels and increased PCNA expression, suggesting a potential protective influence of treatment against age-related inflammation. PCNA-positive nuclei were fewer in the OLD-Con group, moderately distributed in the OLD-25 group, and most abundant in periventricular areas of the OLD-6.25 and ADULT-Con groups. Parameter and treatment group abbreviations: PCNA: Polyclonal Antibody: proliferating cell nuclear antigen polyclonal antibody; IL-1β: interleukin-1 beta; TNF-α: tumor necrosis factor-alpha; OLD-Con: the naturally aged control group; OLD-6.25: the Cannabixir® Medium Flos 6.25 mg/kg naturally aged group; and OLD-25: the Cannabixir® Medium Flos 25 mg/kg naturally aged group; ADULT-Con: the young adult control group. PCNA positive cells quantification in the hippocampal dentate gyrus in different groups. The bars indicate the mean ± SEM. Student t -test: * *p* < 0.05. IL-1β and TNF-α positive cells quantification in the hippocampal dentate gyrus

In parallel, our results highlight that Cannabixir® Medium Flos may offer neuroprotective benefits in naturally aged rodents, potentially through modulation of the ECS. Histological examination of the hippocampus—a region rich in endocannabinoids and CB1 receptors (Glass et al. 1997a, b), revealed significant improvements in the number of neuronal layers in the treatment groups (OLD-6.25 and OLD-25), resembling those of ADULT-Con rats. Additionally, there was a reduction in cells with hyperchromatic and elongated nuclei in the DG region compared to the OLD-Con group (Fig. 6). These observations indicate that this Cannabis strain may positively influence neurogenesis by enhancing neuronal growth and function, likely mediated through ECS modulation. The hippocampus, including its subregions such as DG, CA1, CA2, and CA3, is crucial for cognitive functions and is strongly associated with the onset and progression of age-related neurodegenerative diseases (Giannos and Prokopidis 2022). The ECS, comprising CB1 and CB2 receptors, plays a key role in regulating neurogenesis, synaptic plasticity, and neuroinflammation. Activation of the ECS has been shown to promote neuronal survival and reduce inflammation, processes that are essential in combating the detrimental effects of aging on the brain (Rahimi and Askari, 2023). The structural and functional changes observed in the aging hippocampus (Figs. 7 and 8) are linked to several underlying mechanisms, including neuronal loss, impaired neurogenesis, neuroinflammation, and oxidative stress (Bettio et al. 2017; Giannos and Prokopidis 2022; Hamezah et al. 2017). These mechanisms are interrelated and often trigger cascades of pathological changes as aging progresses. For instance, hippocampal atrophy often accompanies a decline in neurogenesis, which is further compounded by neuroinflammatory responses specific to aging (Bettio et al. 2017). While we hypothesize that THCA-A and CBGA from Cannabixir® Medium Flos could play a role in the observed neuroprotective effects, a comprehensive quantitative analysis of the cannabinoid profile of the product is essential to confirm this hypothesis. The literature on these two compounds is still limited, but some existing studies support this possibility. THCA-A, the acidic precursor to THC and a non-psychotropic cannabinoid, has been suggested to have neuroprotective properties, potentially due to its ability to downregulate pro-inflammatory mediators commonly associated with aging (Nadal et al. 2017). This effect occurs via activation of the peroxisome proliferator-activated receptor gamma (PPAR-gamma**)** and the CB1 cannabinoid receptor (Nadal et al. 2017; Palomares et al. 2020). In addition, CBGA recently demonstrated an anti-inflammatory effect in an acute nephropathy mouse model by suppressing mRNA expression of the cytokines evaluated and a neuroprotective effect in Parkinson’s disease models (Suzuki et al. 2023). Cannabigerol (CBG), the decarboxylated version of CBGA, exerts an antioxidant effect by activating CB2 receptors and inhibits the translocation of nuclear factor-κB (NF-κB), while also modulating the mitogen-activated protein kinase (MAPK) pathway (Carone et al. 2024). These combined actions ultimately lead to the inhibition of cell death. Additionally, CBG has demonstrated an anti-inflammatory effect through downregulation of cytokine activity both at the peripheral and central level (Gugliandolo et al. 2018). Another major compound present in our product is CBD. Schiavon et al. showed that low doses of CBD, between 3 mg/kg and 30 mg/kg led to the improvement of neuronal proliferation (Schiavon et al., 2016). Furthermore, CBD has shown protective effects on the brain that have been linked to its ability to facilitate the survival and differentiation of newborn neurons of the DG (Luján et al., 2019). Based on the current data, we can suggest that this compound shows potential at the neuronal level. However, the mechanisms underlying its influence require further rigorous investigation and robust statistical analysis to be fully understood.

**Fig. 9:**
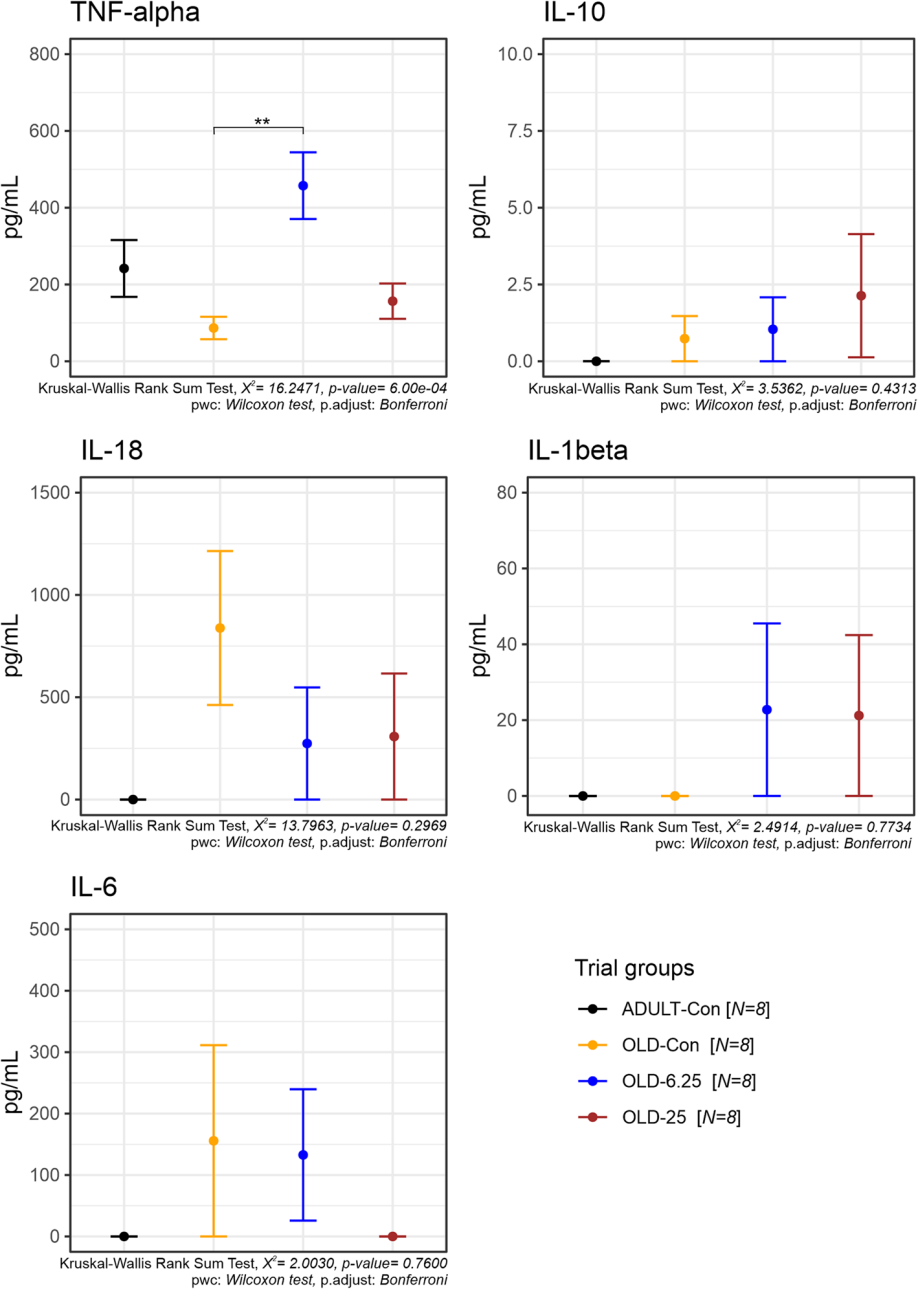
Plasma inflammatory cytokine level variations in treated groups after Cannabixir® Medium Flos therapy. Parameter abbreviations: IL-1β: interleukin-1 beta; TNF-α: tumor necrosis factor-alpha; IL-6: interleukin-6; IL-8: interleukin-8; IL-10: interleukin-10. Data are expressed as group mean ± standard error of the mean (SEM). Treatment group abbreviations: ADULT-Con: the young adult control group; OLD-Con: the naturally aged control group; OLD-6.25: the Cannabixir® Medium Flos 6.25 mg/kg naturally aged group; and OLD-25: the Cannabixir® Medium Flos 25 mg/kg naturally aged group. Significance codes: * *p* < 0.05; ** *p* < 0.01; *** *p* < 0.001

Using GFAP and CD68 immunolabeling as markers of astrocyte and microglia activation, we observed significant immunohistochemical alterations in the hippocampus of treated aged rats compared to the control groups (Fig. 7). While these markers are commonly employed to indicate glial cell activation, they do not provide direct evidence of functional alterations within these cell types. Therefore, although our findings suggest glial activation, further research is necessary to explore the functional consequences of these changes in astrocyte and microglial activity. Focusing on GFAP, a significant increase in astrocyte immunolabeling was noted in the OLD-25 group when compared to both the OLD-Con (*p* = 0.03) and ADULT-Con (*p* = 0.03) groups. The highest average number of GFAP-positive cells was observed in the OLD-25 group (535.50 ± 45.96), while the lowest was recorded in the OLD-Con group (180.50 ± 50.50) (Fig. 7). This suggests an activation of astrocytes in response to treatment, although direct functional changes in astrocyte activity remain unproven. Moreover, we observed a reduction in the number of CD68-marked macrophages in the dentate gyrus and Ammon’s horn of the treated aged rats, closely resembling the histological profile of young adult rats. In contrast, the OLD-Con group exhibited astrocytes appearing as cellular debris or intensely stained with variable sizes, and the number of CD68-positive macrophages was more than three times higher than in the treated groups. The increased number of astrocytes in the treated groups may exert a neuroprotective role by modulating neuroinflammation (Miguel-Hidalgo 2023). Astrocytes can influence the release of pro-inflammatory cytokines, as observed in our study, where the OLD-Con animals displayed higher levels of pro-inflammatory cytokines such as TNF-α (Fig. 9). This modulation by astrocytes helps in promoting the clearance of inflammatory mediators, thereby creating a more favorable microenvironment for neuronal survival (Sofroniew and Vinters 2010). Astrocytes also play a crucial role in regulating extracellular glutamate levels. Activation of the ECS can modulate glutamate uptake and release by astrocytes, preventing excitotoxicity and subsequent neuronal damage (Navarrete and Araque 2008). Cannabinoid receptor activation may induce astrocyte hypertrophy, leading to enhanced astrocytic coverage of synapses and improved synaptic stability, thereby conferring neuroprotection (López et al. 2018; Glass et al., 1997).

In line with previous research, our study indicates that different doses of Cannabixir® Medium Flos exert distinct effects on cell proliferation and apoptosis in aged rats. The PCNA marker revealed perivascular clusters of labeled nuclei, with a mean count of 15.50 ± 3.54, which was significantly lower than in the other groups (*p* < 0.05), indicating reduced cellular proliferation in the OLD-Con group (Fig. 8). Additionally, the low occurrence of caspase-3 positive cells in the OLD-Con group suggests a relatively low level of apoptosis (Fig. 7), although further analysis of cleaved caspase-3 would be necessary to confirm this. These findings are consistent with literature suggesting that cannabinoids can enhance neurogenesis and reduce neuronal apoptosis in aging preclinical models (Schuele et al. 2022; Valeri and Mazzon 2021).

The distribution of PCNA-positive nuclei in the OLD-25 group, which appeared more diffuse, and the low occurrence of caspase-3 positive cells, suggest that increasing the dose does not significantly enhance cell proliferation or apoptosis at this concentration, potentially indicating a plateau effect (Hernandez-Hernandez and Garcia-Fuster 2022; Bilkei-Gorzo et al., 2017)). On the other hand, the observed clusters of PCNA-positive cells around blood vessels and the noticeable reduction in caspase-3 expression in the DG region in the OLD-Con rats suggest that without cannabinoid therapy, there is reduced cell proliferation (Figs. 7 and 8). However, the reduction in caspase-3 expression in this group does not directly indicate increased apoptosis, as further analysis would be needed to assess this relationship more thoroughly. These findings support the role of cannabinoids in mitigating age-related neurodegenerative changes (Martin-Moreno et al. 2012) and emphasize the importance of dose optimization for maximizing therapeutic benefits in aging through cannabinoid administration.

While animal studies provide valuable insights, translating these findings to human contexts requires careful consideration due to species-specific differences and the lack of detailed analyses on cognitive function changes following Cannabis and cannabinoids use. Continued research is necessary to refine dosage strategies, assess the effects of concurrent substance use—especially in individuals with neurodegenerative or chronic pain conditions—and explore modulatory factors such as cognitive reserve and gender differences that may influence how Cannabis and cannabinoids interact with aging.

Although our findings offer important insights, several limitations must be considered when translating our findings to human contexts. One such factor is the exclusive use of male animals in the experiment. Although male rats were chosen to limit variability introduced by hormonal fluctuations, the absence of females in our study may limit the generalizability of the results, given the well-documented sex differences in neurobiology, neuroinflammation, and the effects of cannabinoid treatments (Cooper and Haney 2016). Sex differences are crucial for fully understanding the therapeutic potential of cannabinoids, particularly in age-related conditions. Therefore, future research should include both males and females to compare these sex differences. Behavioral evaluations in animals—such as anxiety, physical performance related to aging frailty, and exploratory behavior—can introduce observer subjectivity, which may affect the accuracy of causality interpretations (McGonigle and Ruggeri 2014). Moreover, significant differences in immune responses, neuroinflammation, and brain structure between rodents and humans can influence research outcomes and limit the direct applicability of findings to human conditions. For example, inflammatory pathways and neurobiological processes can vary substantially between species, impacting the relevance of results obtained from rodent models (Mestas and Hughes 2004). Additionally, species-specific differences in metabolism, genetics, environmental factors, and microbiome play a critical role in determining the pharmacokinetics and pharmacological effects of new drugs. Variations in cannabinoid receptor expression and metabolic enzyme activity between species can affect drug efficacy and safety profiles (Beauchamp et al. 2022; Duffy 2020). These factors underscore the need for cautious interpretation when extrapolating animal models results to human applications. To address these limitations and enhance the translational relevance of our research, future studies should focus on refining dosage strategies and exploring different administration routes. Additionally, investigating the effects of concurrent substance use, particularly in populations with neurodegenerative or chronic pain conditions, will provide a more comprehensive understanding of cannabinoid interactions. Finally, examining factors such as cognitive reserve, gender differences, and individual variability will help elucidate how Cannabis and cannabinoids influence aging and support the development of personalized therapeutic approaches.

## Conclusions

This study provides valuable preliminary data on the effects of Cannabixir® Medium Flos in aging models, focusing on cellular processes such as astrocyte activity, neuroinflammation, cell proliferation, and apoptosis. Our results suggest that different doses of Cannabixir® Medium Flos may modulate these processes, potentially reducing cellular damage and supporting cellular function. Importantly, the lack of observed toxicity at the administered doses supports the safety profile of cannabinoid therapy in aging models.  However, these observations highlight the need for more robust studies to confirm the therapeutic potential of cannabinoids in age-related neurodegenerative conditions and to further explore the underlying mechanisms of their effects on aging and neurodegeneration. Targeting the ECS could be a promising strategy for developing therapies aimed at promoting healthy aging and longevity. 

## Data Availability

No datasets were generated or analysed during the current study.

## Abbreviations

AD: Alzheimer’s disease
BA: Basophils
CBGA: Cannabigerolic acid
CBD: Cannabidiol
CBG: Cannabigerol
CD68: Microglial activation marker
COX-2: Cyclooxygenase 2
DG: Dentate gyrus
ECS: Endocannabinoid system
EO: Eosinophils
EPM: Elevated plus maze
GFAP: Glial fibrillary acidic protein
Hb: Hemoglobin
HCT: Hematocrit
IHC: Immunohistochemical analysis
IL-1β: Interleukin-1β
IL-6: Interleukin-6
IL-8: Interleukin-8
IL-10: Interleukin-10
LY: Lymphocyte
MCH: Mean corpuscular hemoglobin
MCHC: Mean corpuscular hemoglobin concentration
MCV: Mean corpuscular volume
MPV: Mean platelet volume
MO: Monocyte
NE: Neutrophils
PPAR-gamma: Peroxisome proliferator-activated receptor gamma
PLT: Platelet count
PCNA: Proliferating cell nuclear antigen
RBC: Red blood cell count
RDW: Red cell distribution width
SGZ: Subgranular zone
THC: ∆9-Tetrahydrocannabinol
THCA-A: ∆9-Tetrahydrocannabinolic acid
TNF-α: Tumor necrosis factor-alpha
WBC: White blood cell count
